# The Somogyi hypothesis: a parallelism with Michael Somogyi’s life

**DOI:** 10.1007/s42000-024-00624-0

**Published:** 2024-12-24

**Authors:** Tomás González-Vidal, Jessica Ares-Blanco, Elías Delgado, Edelmiro Menéndez-Torre

**Affiliations:** 1 Department of Endocrinology and Nutrition, Hospital Universitario Central de Asturias/University of Oviedo, Oviedo, Spain; 2 Instituto de Investigación Sanitaria del Principado de Asturias (ISPA), Oviedo, Spain; 3 Department of Medicine, University of Oviedo, Oviedo, Spain; 4 Centre for Biomedical Network Research on Rare Diseases (CIBERER), Instituto de Salud Carlos III, Madrid, Spain

**Keywords:** Somogyi, Hypoglycemia, Insulin, Diabetes

## Abstract

Michael Somogyi (Somogyi Mihály, 1883–1971) was a Hungarian biochemist who developed his scientific career in Europe and, primarily, the United States. He gave the name to the eponymous Somogyi effect or Somogyi hypothesis (in short, rebound hyperglycemia after insulin-induced hypoglycemia, particularly nocturnal), which was an axiom in the treatment of diabetes for decades. Although it is currently debated whether the Somogyi hypothesis is a real or relevant phenomenon in patients with diabetes, Somogyi's other significant career achievements are often overlooked. The aim of this historical note is to compile and highlight Michael Somogyi's scientific achievements. Michael Somogyi was a pioneer in the administration of insulin to patients with diabetes in the United States and in devising a method for insulin production. In addition, he highlighted the relevance of diet in patients with diabetes and was one of the first chemists to be integrated into clinical laboratories. There, Somogyi standardized long-lasting biological determinations, such as that of amylase, and he was one of the first scientists to combine basic research (from his training as a biochemist) with clinical research in close collaboration with physicians caring for patients, which is what we know today as translational research. Notably, the trajectory of his scientific career resembles the rebound effect of Somogyi's hypothesis: after reaching a low point of work activity well below his professional qualifications, his effort and tenacity led to the aforementioned achievements, and he became part of the history of hypoglycemia and diabetes.

## Introduction

**Fig. 1:**
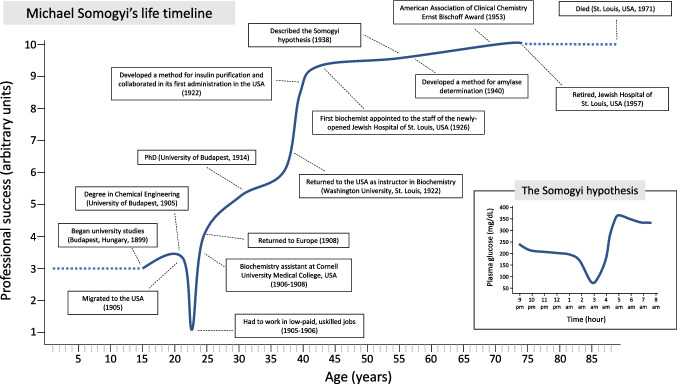
Schematic representation of Dr. Somogyi’s biography with the main events of his professional career. The units of the ordinate axis are arbitrary and attempt to represent the professional category of the work carried out at each moment in relation to his academic training. The lows and highs of this indicator resemble the ups and downs that characterize the so-called Somogyi effect that he described (lower panel)

Michael Somogyi (Somogyi Mihály, 1883–1971) was born in the village of Zsámánd, which at the time was part of the Austro-Hungarian Empire. Following the Treaty of Trianon in 1920, this small village of only 174 inhabitants (as of January 2021), just 1 kilometer from the border with Hungary, now belongs to Austria and is known as Reinersdorf [1]. For all intents and purposes, however, Somogyi is considered Hungarian [2–5]. Although the Reinersdorf website identifies him as a prominent local figure [1], there is currently no monument in the village commemorating Somogyi. Nevertheless, a monument in the village honors its many inhabitants who emigrated to the United States by the early twentieth century, as was the case with Somogyi. This historical note aims to recall Somogyi’s life and the highlights of his scientific career.

## Courage and ambition to face tough beginnings

Somogyi was one of eight children of the local butcher and storekeeper with the original surname Steiner, which he changed to Somogyi in 1904 [2]. When Somogyi was nine years old, his father died and his family fell on hard times [6]; however, this did not stop him in his quest for knowledge. At the age of 16, he enrolled in the Faculty of Chemistry at Eötvös Loránd Budapest University of Technology, working his way through school without family help and obtaining a degree in chemical engineering in 1905 [2–4, 6]. Throughout his undergraduate days he subsisted on a diet of rice, beans, and milk, with very little meat [6]. This was his first personal experience of food scarcity, which sparked his interest in the effects of food on body chemistry.

The autocratic Austro-Hungarian Empire did not suit young Somogyi, who sought inspiration in romantic books about the United States [2, 3, 6, 7]. After working as a biochemistry assistant for another 6 months, he moved to the United States without any knowledge of English or any certificate to prove his advanced studies. He even refused letters of introduction to American scientists from Professor Franz Tangl, a Hungarian physiologist, stating that “all that is unnecessary in the United States” [6]. Despite what he had read in books, he soon realized that America was more a promised land for manual labor than for intellectual work [4]: the only employment he could find was driving a horse and buggy for a German physician in New York [6, 7]. After saving some money, Somogyi bought a rail ticket to Cincinnati, the farthest point west he could afford [6]. In Cincinnati, he worked at a rope factory for a weekly wage of $7 [7] (Fig. 1). He shared his room with two radical Russian laborers, who reproached him for his eagerness to work and for keeping the light on in the room at night to study English. His weekly budget for English lessons, bathing, and food was $2 [6]. This was the second time in his life during which Somogyi had the opportunity to personally experience the physical effects of food shortage.

He eventually wrote to Professor Tangl in Budapest to ask for the previously scorned letters of introduction. After obtaining his letters of recommendation and study certificates, Somogyi applied for a job as a laboratory assistant at Bellevue Hospital in New York. During his interview with Dr. Graham Lusk, a distinguished physiologist, Somogyi expressed his willingness to accept any position, even that of cleaning the laboratory [6]. With Dr. Lusk's assistance, Somogyi secured a position as a research assistant in biochemistry at Cornell University Medical College (Fig. 1). He worked at Cornell from 1906 to 1908, saving most of his salary to buy a ticket back to Europe. Upon returning home, his mother convinced him to stay [6]. He remained, married his love, (née) Erzsébet (Elizabeth) von Gross [2], and started a family.

## Settling in the United States: the beginnings of insulin extraction and administration

**Fig. 2:**
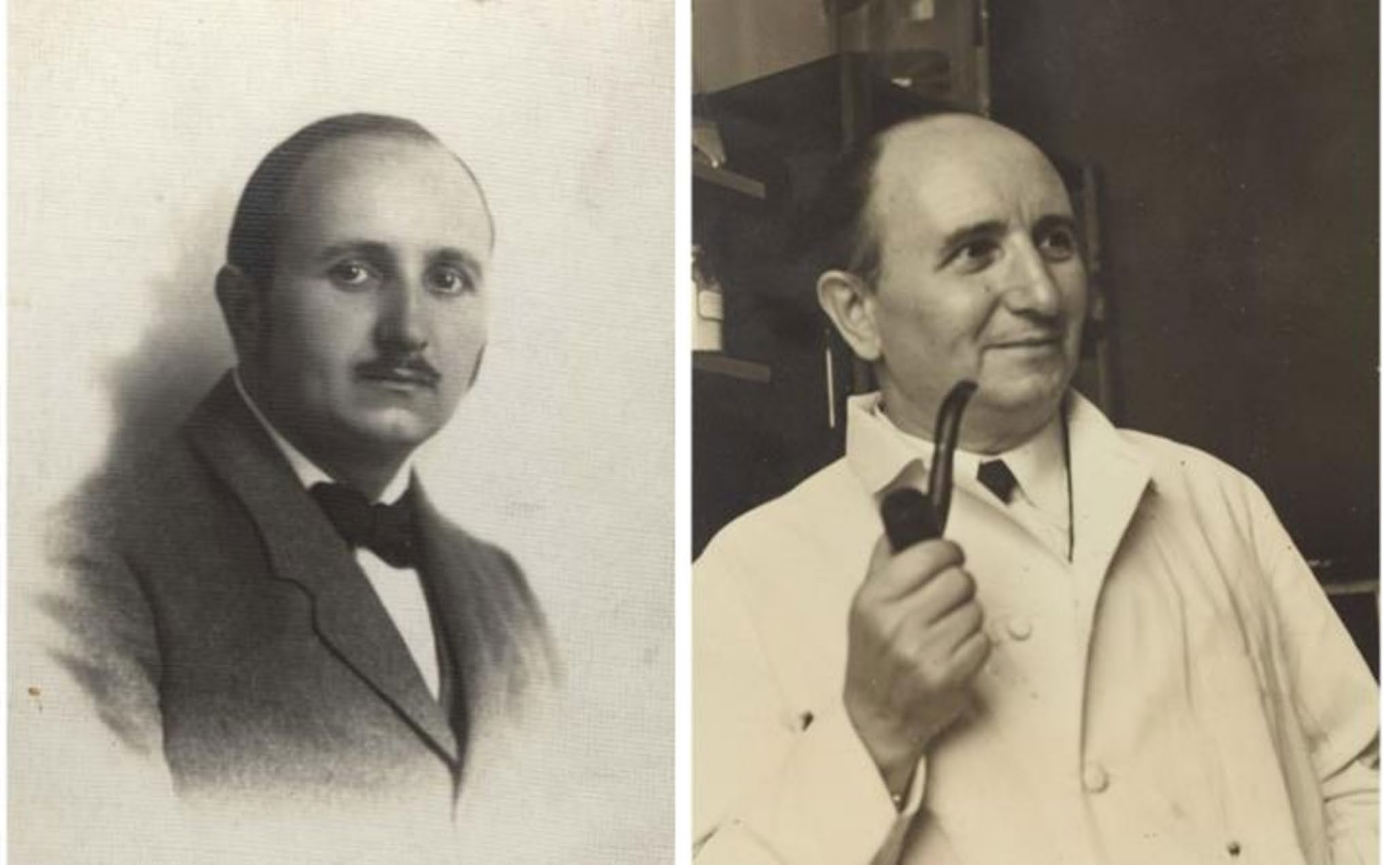
Portraits of Dr. Somogyi during his time in Hungary (left photo, the back of which reads "Taken in Europe", c. 1900–1925) and during his later time at the Jewish Hospital in St. Louis (MO, USA; right photo, c. 1940–1949). Courtesy of the Science History Institute, Philadelphia, USA. Available at: https://digital.sciencehistory.org. No known copyright (left photo); In Copyright-Rights-holder(s) unlocatable or unidentifiable (right photo)

In Budapest (Fig. 2), he served as the head chemist of the Municipal Laboratory for more than a decade and obtained his PhD in Biochemistry from the University of Budapest in 1914, submitting a dissertation on catalytic hydrogenation (*A katalysises uton hydrogennel telittet zsirok vizsgálata*; lit. Examination of hydrogen-saturated fats after catalysis) (Fig. 1). During World War I (1914–1918), Somogyi was responsible for providing food to the destitute from a model central kitchen and various distribution centers, in an attempt to keep 40000 people alive with pickled turnips, sauerkraut, and a small amount of flour [6, 7]. Again, Somogyi had to deal with the consequences of food scarcity, which would further shape his future interest in the effects of diet on metabolic disorders such as diabetes.

After having lived in the United States, Somogyi found Europe to be feudal. “The attendant in the government laboratory always came to attention with a click of the heels and a stiff bow when he asked Somogyi what he wanted for a mid-morning snack" [6]. Hungary experienced a turbulent political period after World War I, and Somogyi had to move to nearby Austria with his family. In 1922, he returned to the United States at the request of his former colleague at Cornell University, Philip A. Schaffer [2, 3]. He then became an instructor of biochemistry at the medical school of Washington University in St. Louis, where he collaborated with P.A. Schaffer and Edward A. Doisy (the Nobel Prize-winning discoverer of vitamin K) on a method for extracting insulin from the pancreas of dogs by means of isoelectric precipitation (Table 1) [8, 9]. Credit for the discovery of insulin extraction is disputed. The method for its purification with successful therapeutic utility is most commonly attributed to Frederick Banting, John Macleod, Charles Best, and James Collip at the University of Toronto in Canada (1921), for which the first two were awarded the Nobel Prize [10, 11]. Some of Somogyi's colleagues would later acknowledge that Washington University’s insulin extraction had preceded that of the Canadian group, but that P. A. Schaffer himself, Somogyi's mentor and dean of the faculty of medicine, delayed its publication [2, 12]. At any rate, in early October 1922, almost simultaneously with the Canadian discoverers, an 18-month-old baby boy at St. Louis Children's Hospital became the first person with diabetes in the United States to be treated with insulin (Fig. 1, Table 1). Insulin offered hope for extending the lives of children with diabetes, who at that time typically lived no more than a few months or years. Somogyi, a biochemist without a medical degree, became interested in diabetes for the rest of his life. He worked closely with clinicians and contributed to the care of over 5000 patients with diabetes during his lifetime [6, 7].

**Table 1:** Main scientific contributions of Michael Somogyi

Scientific contribution	Date
**Contributions to the pathophysiology of insulin and counterregulatory hormones**	
Co-worked in the development of a method to purify insulin from dog pancreases	1922
Participated in the team that treated the first child with diabetes with insulin in the United States	1922
Issued the hypothesis of post-hypoglycemic hyperglycemia (the Somogyi effect)	1938
Proposed diet and weight loss over insulin therapy as the key to controlling diabetes in people with overweight	c. 1947–1949
Optimized nocturnal glycemic control by splitting the nighttime insulin dose	1959
**Contributions to clinical biochemistry**	
Second chemist to be in charge of a clinical laboratory in a United States hospital	1926
Standardized routine laboratory determinations of glucose, ketone bodies, glycogen and potassium	c. 1926–1957
Developed urine glucose determination devices for clinical use in people with diabetes	c. 1930–1950
Developed a standardized method for amylase determination which was the reference for decades (Somogyi units)	1940

## A translational researcher: urine glucose meters and amylase Somogyi units

**Fig. 3:**
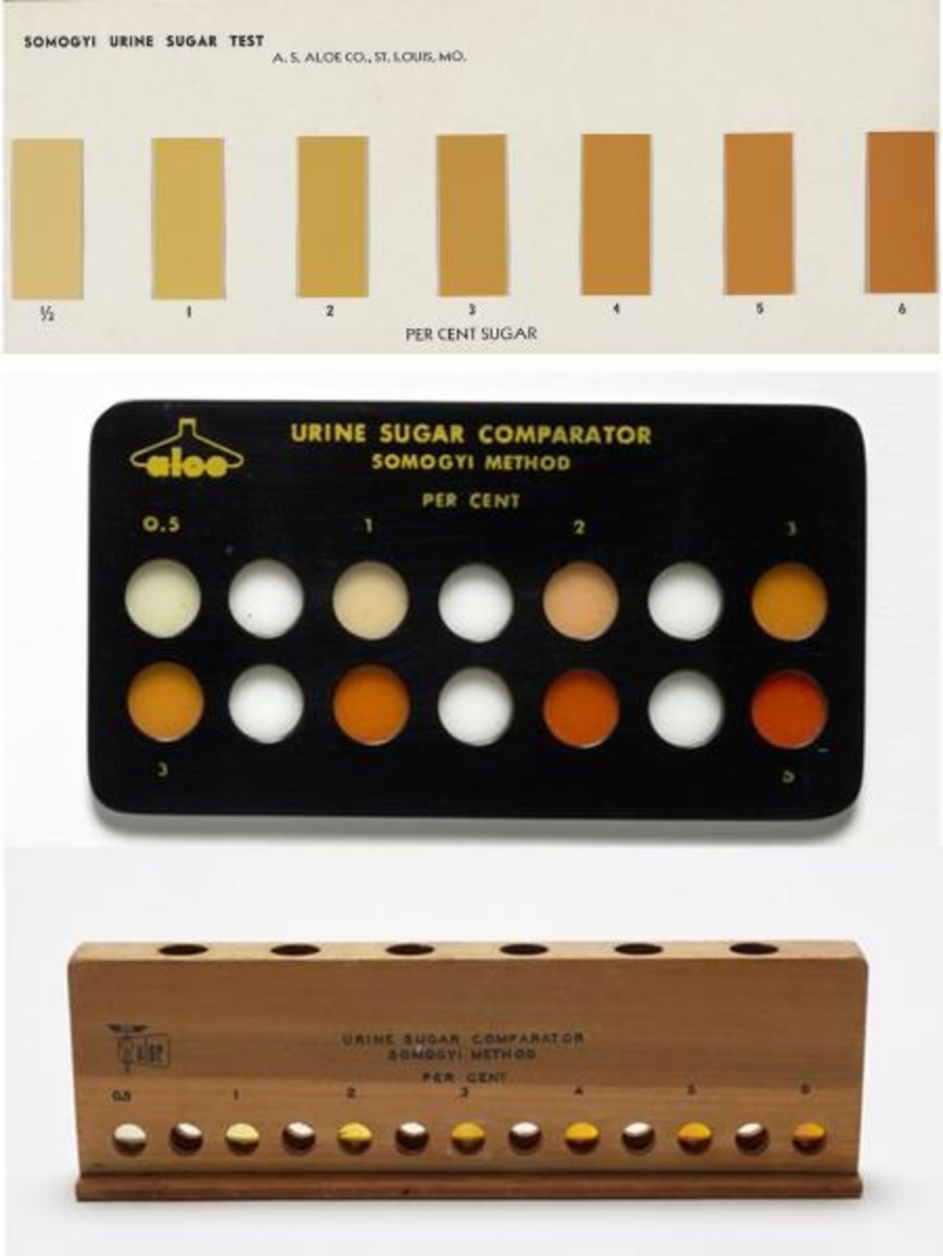
Various devices for estimating urinary glucose designed by Dr. Somogyi (c. 1930–1950) and developed by the local A.S. Aloe Co. in St. Louis (MO, USA). The estimation of urinary glucose concentration was carried out by colorimetric comparison, on paper (upper panel), plastic wells (middle panel), or wooden comparator for urine in a test tube (lower panel). Courtesy of the Science History Institute, Philadelphia, USA. Available at https://digital.sciencehistory.org. Public Domain Mark 1.0 (upper panel); Creative Commons Attribution 4.0 International License (middle and lower panel)

In 1926, Somogyi became the first biochemist to join the staff of the newly established Jewish Hospital of St. Louis (Fig. 2) [13]. This hospital was the second in the USA to employ a full-time chemist in its laboratories (Table 1). At that time, before the advent of molecular biology, individuals who transitioned from other professions to medicine often remained outsiders for a prolonged period [4]; however, Somogyi was able to integrate well with the other clinicians. He established a laboratory of Clinical Chemistry, initially a “test tube and flask” laboratory with a total budget of $1,200 [6, 13]. He and his first assistant, Hildegard Kramer, developed approximately 12 tests, with sugar and non-protein nitrogen estimation being the most commonly performed [6, 13]. Somogyi contributed to the standardization of the measurement of ketone bodies, glycogen, and potassium (Table 1) [7]. He also developed a cost-effective method for screening for diabetes and monitoring its control in urine by using sodium carbonate and heat (Table 1). This development led to the creation of popular tests, including various types of urine sugar comparators (Fig. 3). In 1940, he developed a method for determining serum and urine amylase (diastase), a well-known marker of pancreatitis (Fig. 1, Table 1). The so-called Somogyi units (abbreviated, SU) are a measure of the hydrolyzing action of serum amylase on starch. A Somogyi unit is equivalent to the amount of enzyme in 100 ml of sample required to produce 1 mg of glucose when acting on a standard starch solution under defined conditions of time, pH, and temperature [14–16]. These units became reference units in the clinical laboratory for decades, as stated in textbooks [17], and they were still in use at the beginning of the twenty-first century [18, 19]. Although Somogyi units have widely been replaced by International Units, the American Board of Internal Medicine included their equivalence with Somogyi units in their 2024 list of reference ranges [20].

## The Somogyi hypothesis

Somogyi is best known for his hypothesis that excessive insulin treatment could destabilize diabetes. He presented this hypothesis at a meeting of the Medical Society in St. Louis in 1938 under the theme “Observation on Unmanageable Patients”, and later published it in the Weekly Bulletin of the Society [21, 22]. The theory, known as the “Somogyi effect” or “Somogyi phenomenon,” suggests that “hypoglycemia begets hyperglycemia”. According to Somogyi's hypothesis, insulin-induced hypoglycemia at night would stimulate the secretion of counterregulatory hormones [23–25] or would induce eating [26], resulting in hyperglycemia the following morning (Fig. 1, Table 1). The notion spread quickly among doctors and patients with diabetes, so that the surname Somogyi became a popular verb, as in “Do you Somogyi?”: the doctor asks patients if, for example, they suspect nocturnal hypoglycemia in the context of consistently elevated morning blood glucose levels [27]. The Somogyi phenomenon (also known as rebound hyperglycemia or post-hypoglycemic hyperglycemia) was widely accepted in clinical practice and became integrated into clinical teaching for decades. The 14th edition of Harrison's Principles of Internal Medicine (1998) still devoted attention to it in the management of patients with diabetes [28]. However, its mechanisms were later questioned [26, 29]. Although there were exceptions [30], in most experimental [31, 32] and observational [33, 34] studies, fasting blood glucose was not found to be elevated after nocturnal hypoglycemia, contrary to what Somogyi suggested. Moreover, other mechanisms for morning hyperglycemia are well known, such as the dawn phenomenon: morning hyperglycemia that is not preceded by hypoglycemia, caused by hypoinsulinemia or by insulin resistance due to a physiological increase in growth hormone (at night) and cortisol (at dawn) [35–37]. All in all, recent reviews have concluded that there is insufficient evidence to support the use of the Somogyi phenomenon as a standard precaution in practice [38]. Somogyi's experiments were painstaking, manually performed by studying a large number of glycosurias in hundreds of patients. One can only imagine the possible discoveries of someone with such a capacity for work if he had at his disposal, as today, continuous interstitial glucose monitoring systems.

## Additional contributions to diabetes treatment: diet and insulin dosing

The aforementioned forced experiences with diet throughout his life forged in Somogyi an interest in the effects of diet on health, particularly in patients with diabetes [39–44]. At a time when the distinction between type 1 and type 2 diabetes was not yet widely understood, he argued that a combination of diet and weight loss could effectively manage the condition for many patients with diabetes (Table 1) [7]. In particular, he sensed that insulin treatment might not be necessary for patients with diabetes and overweight, given that the conditions could be controlled with diet and weight loss [7]. Today, weight loss through diet and physical exercise is the first-line intervention in patients with type 2 diabetes, overweight, and insulin resistance. Somogyi told his patients, “When you were born, the Lord put aside so much food for your lifetime. Why are you in such a hurry to eat up your share?” and “Yes, you may put a little margarine on your bread, but remember, you belong to the varnisher’s union, not the plasterer’s” [6]. Furthermore, to improve nocturnal glycemic control, Somogyi proposed, probably for the first time, to split the evening insulin dose into two fractions: a first dose before dinner and a second dose before bedtime (Table 1). Similar to the basal-bolus regimens available today and unimaginable at that time, the first dose would cover the dinner intake and the second dose would mimic the nocturnal basal secretion of pancreatic insulin [3, 22].

## Retirement: father of a family

Somogyi held the position of first biochemist at The Jewish Hospital of St. Louis until 1953, when he was named biochemist emeritus at the institution [13]. That year, he was granted the Ernst Bischoff American Association of Clinical Chemistry Award. In 1964, he would also receive the Donal D. Van Slyke Award. He used to say that “The medals did not make me grow any higher in my own eyes. The pleasure of knowing my work has been useful – that has been important” [3]. He retired in 1957 (Fig. 1) and continued to live in St. Louis with his family, where he gladly welcomed visiting scientists from his home country [4]. His first wife, Erzsébet, whom he had married in Hungary, died a few years after returning to the USA. His second wife, whom he married in the USA, was also named Erzsébet (maiden name, Simonyi) and was also of Hungarian descent [6]. Somogyi became the father of a large family: he had six children (Agnes, Erwin, and the twins Victor and Robert from his first wife; and Anna Li and Susan from his second wife), nine grandchildren, and 15 great-grandchildren. Although none of his descendants chose to study medicine, several of them developed relevant scientific careers in the United States. His son Erwin followed in his footsteps and became a chemical engineer. His grandson, Michael Stark, worked as a software engineer for NASA. Today, some of his great-grandchildren and great-great-grandchildren are practicing and studying scientific careers such as environmental science, software engineering, and aerospace engineering.

Somogyi suffered a stroke at the age of 86 and died in St. Louis in 1971 at the age of 88. The Michael Somogyi Diabetes Foundation was later established by his close colleagues and friends to maintain his legacy; unfortunately, however, the foundation has ceased its activity. In 2004, the Hungarian Diabetes Association established the Somogyi Award to honor individuals who have made significant contributions to the understanding of hypoglycemia and its counter-regulatory mechanisms [45].

## Conclusions and legacy

Somogyi is, after his hypothesis, one of the two eponyms recorded in medicine in relation to hypoglycemia, along with Whipple (after Allen Oldfather Whipple for his famous triad indicative of endogenous hyperinsulinism) [46]. It is possible, as mentioned above, that the hypothesis that made him most famous is not as real in practice as was thought at the time. As his second wife remembered in an interview conducted after his death [6], one of Somogyi’s favorite sayings was “The trouble with some people is not what they know, but what they know and it ain’t so”. It is a frequent consequence of scientific progress. However, one cannot forget the many accomplishments of his career: he pioneered the administration of insulin to patients with diabetes and devised a method for its commercial production; he emphasized the importance and the effects of diet in patients with diabetes; he was one of the first chemists to be integrated into clinical laboratories where he standardized long-lasting biological determinations, such as that of amylase; and lastly, he was one of the first scientists to combine basic research and clinical research in close collaboration with clinicians who cared for patients, what is known today as translational research. Notably, the trajectory of his life, as depicted in Fig. 1, resembles the rebound effect of Somogyi's hypothesis: after hitting a low point working in conditions far below his professional qualification, he was not only able to pick himself up, but to reject comfortable positions until achieving the milestones mentioned in the previous paragraphs. In that respect, Somogyi's scientific career is an example of intellectual restlessness, endurance, and determination.

## Data Availability

Not applicable.

## References

[1] Anonymous (2024) Südburgenland erleben. https://www.sued-burgenland.com/reinersdorf.htm. Accessed August 28 2024

[2] Soltész G (2022) Somogyi Mihály (1883–1971). Diabetologia Hungarica 30:223–226

[3] Soltész G (2002) Michael Somogyi. The man behind “the phenomenon.” Diabetologia Hungarica 10(Suppl 2):5–7

[4] Szállási A (2001) Egy ismert effektus alig ismert névadója: Somogyi Mihály (1883–1971) [A hardly known eponym of a well-known effect: Mihály Somogyi (1883–1971)]. Orv Hetil 142:1749–1750 11570011

[5] Jermendy G (2002) Somogyi emléke a diabetológiában [Memory of Dr. Somogyi in diabetology]. Orv Hetil 143:532 11963410

[6] Skinner O (1971) He gave diabetics new life. St Louis Post-Dispatch 1971 (6 oct) page 72. https://www.newspapers.com/newspage/140664078/. Accessed August 28 2024

[7] Walker H (1972) Michael Somogyi, PhD (1833–1971). Clin Chem 17:1138

[8] Somogyi M, Doisy EA, Schaffer PA (1924) On the preparation of insulin. J Biol Chem 60:31–38

[9] Doisy EA, Somogyi M, Shafer PA (1923) Some properties of an active constituent of pancreas (insulin). J Biol Chem 55:31–32

[10] de Leiva-Hidalgo A, de Leiva-Pérez A (2023) On the occasion of the centennial of insulin therapy (1922–2022), II-Organotherapy of diabetes mellitus (1906–1923): Acomatol. Pancreina Insulin Acta Diabetol 60:163–189 36585966 10.1007/s00592-022-02014-7 PMC9852216

[11] Lambert C, Delgado E (2024) 100 years since the discovery of insulin, from its discovery to the insulins of the future. Biomedicines 12:533 38540146 10.3390/biomedicines12030533 PMC10968222

[12] Anonymous transcript (1982) Harry Agress interview. Washington School of Medicine Oral History Project. http://beckerexhibits.wustl.edu/oral/transcripts/agress.html. Accessed August 28 2024

[13] Anonymous (1967) Clinical laboratories: A rich history and an expanding future. 216 – A community publication of the Jewish Hospital of St. Louis 16(7):3. https://digitalcommons.wustl.edu/cgi/viewcontent.cgi?article=1087&context=bjc_216. Accessed August 28 2024

[14] Somogyi M (1938) Micromethods for the estimation of diastase. J Biol Chem 125:399–414

[15] Somogyi M (1941) Diastatic activity of human blood. Arch Intern Med (Chic) 67:665–679

[16] Somogyi M (1960) Modifications of two methods for the assay of amylase. Clin Chem 6:23–35 13832788

[17] Greenberger NJ, Toskes PP, Isselbacher KJ (1983) Diseases of the pancreas. In: Petersdorf RG, Adams RD, Braunwald E, Isselbacher KJ, Martin JB, Wilson JD (eds) Harrison’s Principles of Internal Medicine, 10th edn. McGraw-Hill, New York, pp 1836–1848

[18] Kaman L, Behera A, Singh R, Katariya RN (2001) Internal pancreatic fistulas with pancreatic ascites and pancreatic pleural effusions: recognition and management. ANZ J Surg 71:221–225 11355730 10.1046/j.1440-1622.2001.02077.x

[19] Xu ZR, Hu CH, Xia MS, Zhan XA, Wang MQ (2003) Effects of dietary fructooligosaccharide on digestive enzyme activities, intestinal microflora and morphology of male broilers. Poult Sci 82:1030–1036 12817461 10.1093/ps/82.6.1030

[20] American Board of Internal Medicine (2024) ABIM Laboratory Test Reference Ranges. https://www.abim.org/Media/bfijryql/laboratory-reference-ranges.pdf. Accessed August 28 2024

[21] Somogyi M, Kirstein M (1938) Insulin as a cause of extreme hyperglycemia and instability. Wkly Bull St Louis Med Soc 32:498–510

[22] Somogyi M (1959) Exacerbation of diabetes by excess insulin action. Am J Med 26:169–191 13617275 10.1016/0002-9343(59)90307-9

[23] Somogyi M (1950) Studies of arteriovenous differences in blood sugar. V. Effect of epinephrine on the rate of glucose assimilation. J Biol Chem 186:513–526 14794646

[24] Somogyi M (1951) Mechanism of epinephrine-hyperglycemia. Endocrinology 49:774–781 14906311 10.1210/endo-49-6-774

[25] Bolli GB, Gottesman IS, Campbell PJ, Haymond MW, Cryer PE, Gerich JE (1984) Glucose counterregulation and waning of insulin in the Somogyi phenomenon (posthypoglycemic hyperglycemia). N Engl J Med 311:1214–1219 6387483 10.1056/NEJM198411083111904

[26] Raskin P (1984) The Somogyi phenomenon. Sacred cow or bull? Arch Intern Med 144:781–787 6370162

[27] Soltész G (2011) 'Do you Somogyi' - 40 éve halt meg Somogyi Mihály (1883–1971). https://www.doki.net/tarsasag/diabetes/hirek.aspx?&nid=19138&cid=277. Accessed August 28 2024

[28] Foster DW (1998) Diabetes mellitus. In: Fauci AS, Braunwald E, Isselbacher KJ, Wilson JD, Martin JB, Kasper DL, Hauser S, Longo DL (eds) Harrison’s Principles of Internal Medicine, 14th edn. McGraw-Hill, New York, pp 2060–2081

[29] Gale EA, Kurtz AB, Tattersall RB (1980) In search of the Somogyi effect. Lancet 2:279–282 6105438 10.1016/s0140-6736(80)90233-0

[30] Perriello G, De Feo P, Torlone E, Calcinaro F, Ventura MM, Basta G, Santeusanio F, Brunetti P, Gerich JE, Bolli GB (1988) The effect of asymptomatic nocturnal hypoglycemia on glycemic control in diabetes mellitus. N Engl J Med 319:1233–1239 3054544 10.1056/NEJM198811103191901

[31] Tordjman KM, Havlin CE, Levandoski LA, White NH, Santiago JV, Cryer PE (1987) Failure of nocturnal hypoglycemia to cause fasting hyperglycemia in patients with insulin-dependent diabetes mellitus. N Engl J Med 317:1552–1559 3317053 10.1056/NEJM198712173172502

[32] Hirsch IB, Smith LJ, Havlin CE, Shah SD, Clutter WE, Cryer PE (1990) Failure of nocturnal hypoglycemia to cause daytime hyperglycemia in patients with IDDM. Diabetes Care 13:133–142 2190769 10.2337/diacare.13.2.133

[33] Choudhary P, Davies C, Emery CJ, Heller SR (2013) Do high fasting glucose levels suggest nocturnal hypoglycaemia? The Somogyi effect-more fiction than fact? Diabet Med 30:914–917 23672623 10.1111/dme.12175

[34] Huang Y, Lou X, Huang W, Qiu J, Jiang C, Sun J, Tao X (2022) Confirmation of the absence of Somogyi effect in patients with type 2 diabetes by retrospective continuous glucose monitoring systems. Int J Endocrinol 2022:6599379 36237834 10.1155/2022/6599379 PMC9553369

[35] Rybicka M, Krysiak R, Okopień B (2011) The dawn phenomenon and the Somogyi effect - two phenomena of morning hyperglycaemia. Endokrynol Pol 62:276–284 21717414

[36] Perriello G, De Feo P, Torlone E, Fanelli C, Santeusanio F, Brunetti P, Bolli GB (1990) Nocturnal spikes of growth hormone secretion cause the dawn phenomenon in type 1 (insulin-dependent) diabetes mellitus by decreasing hepatic (and extrahepatic) sensitivity to insulin in the absence of insulin waning. Diabetologia 33:52–59 2406181 10.1007/BF00586461

[37] Bolli GB, De Feo P, De Cosmo S, Perriello G, Ventura MM, Calcinaro F, Lolli C, Campbell P, Brunetti P, Gerich JE (1984) Demonstration of a dawn phenomenon in normal human volunteers. Diabetes 33:1150–1153 6389230 10.2337/diab.33.12.1150

[38] Reyhanoglu G, Rehman A (2023) Somogyi Phenomenon. In: StatPearls [Internet]. Treasure Island (FL): StatPearls Publishing 31855369

[39] Somogyi M, Cook RJ (1947) Effect of the fat content of diets on blood sugar. Proc Soc Exp Biol Med 65:336–339 20250480 10.3181/00379727-65-15953

[40] Pareira MD, Somogyi M (1948) Rationale of parenteral glucose feeding in the postoperative state. Ann Surg 127:417–42518907433

[41] Somogyi M (1948) A paradoxical effect of insulin on glucose assimilation. Fed Proc 7:190 18938813

[42] Somogyi M (1948) Studies of arteriovenous differences in blood sugar; effect of alimentary hyperglycemia on the rate of extrahepatic glucose assimilation. J Biol Chem 174:189–200 18914074

[43] Somogyi M (1949) Studies of arteriovenous differences in blood sugar; effect of intravenous insulin and simultaneous glucose feeding. J Biol Chem 179:1289–1297 18134591

[44] Somogyi M, Goldwasser HV (1959) Quantitative relationship between insulin dosage and amount of carbohydrates utilized in diabetic persons. Am J Med 26:165–168 13617274 10.1016/0002-9343(59)90306-7

[45] Magyar Diabetes Társaság (Hungarian Diabetes Association) (2024) Somogyi Award. https://www.doki.net/tarsasag/diabetes_bak/info.aspx?sp=34&web_id. Accessed August 28 2024

[46] González-Vidal T, Delgado E, Menéndez-Torre E (2023) Whipple of Whipple’s triad. J Clin Pract Res 45:655–657 10.14744/cpr.2023.03511 PMC12478724 41256744

